# Complete Genome Sequence of Cellulomonas sp. Strain Y8, a High-GC-Content Plasmid-Free Heavy Metal-Resistant Bacterium Isolated from Farmland Soil

**DOI:** 10.1128/MRA.01066-19

**Published:** 2019-11-14

**Authors:** Jinghao Chen, Chao Xing, Xin Zheng, Xiaofang Li

**Affiliations:** a University of Chinese Academy of Sciences, Beijing, China; b Key Laboratory of Agricultural Water Resources, Hebei Key Laboratory of Soil Ecology, Center for Agricultural Resources Research, Institute of Genetics and Developmental Biology, Chinese Academy of Sciences, Shijiazhuang, China; University of Maryland School of Medicine

## Abstract

We report the complete genome sequence of cadmium-resistant Cellulomonas sp. strain Y8, isolated from farmland soil. The 4.5-Mbp genome contains 4,074 genes, with an approximate GC content of 75%. This work might help in understanding how strain Y8 survives under heavy metal stress.

## ANNOUNCEMENT

Cellulomonas sp. strain Y8, which has strong cadmium (Cd) resistance, was isolated from farmland soil of the Agro-Ecosystem Experimental Station located in Luancheng, Shijiazhuang, China (37°53′N, 114°41′E), in 2017. Colonies of Y8 appear as smooth, opaque, pale-yellow, moist spheres. Y8 had a high growth rate on a Luria-Bertani plate at 37°C under aerobic conditions, and no significant growth inhibition was observed when it was inoculated on a Luria-Bertani plate containing 2 mM CdCl_2_. It was therefore desirable to obtain the genomic sequence of the *Cellulomonas* strain that was able to thrive under Cd stress.

Genomic DNA was extracted from Y8 by using the PureLink Pro 96 genomic DNA purification kit (Thermo Fisher, USA), following the standard instructions. As the template, the 16S rRNA gene was then amplified and sequenced to verify the quality of the genomic DNA. The complete genome of *Cellulomonas* sp. strain Y8 was sequenced by using both the Illumina HiSeq (USA) and PacBio RS II (USA) platforms, according to standard protocols (1). For next-generation sequencing, the library preparations were constructed following the manufacturer’s protocol. For each sample, 100 ng genomic DNA was randomly fragmented to <500 bp by sonication (Covaris S220). The fragments were treated with end prep enzyme mix. Size selection of adaptor-ligated DNA was performed, and then fragments of ∼470 bp (with an approximate insert size of 350 bp) were recovered. Each sample was then amplified by PCR for 8 cycles using the P5 and P7 primers. The PCR products were cleaned up and validated using an Agilent 2100 Bioanalyzer (USA) and quantified with a Qubit 3.0 fluorometer (Invitrogen, USA). Then libraries with different indices were multiplexed and loaded onto an Illumina HiSeq instrument according to the manufacturer’s instructions. Cutadapt 1.9.1 (2) was employed to control the quality of the pass filter data, and reads with base groups having a quality score below 20 at both ends, as well as sequences containing more than 10% N bases or those that were less than 75 bp in length, were removed.

For PacBio sequencing, the genomic DNA was sheared, and 10-kb double-stranded DNA fragments were selected. The DNA fragments were end repaired and ligated with universal hairpin adapters. The library was sequenced in a PacBio RS II instrument (1, 3). The PacBio reads were assembled using Falcon with wgs-assembler 8.2 (4–6). Then, the genome was recorrected with Pilon 1.22 (7) using Illumina data (SRA accession number SRR9639642) or with Quiver using PacBio reads (SRA accession number SRR9639643). The GC content was calculated by using an in-house Perl script. Prodigal gene-finding software was used to identity coding genes (8). Transfer RNAs were detected in the genome by using tRNAscan-SE (9). rRNAs were identified by using RNAmmer (10). Default parameters were used except where otherwise noted. Protein-coding genes were assigned using BLASTp against the following databases: the Reference Sequence nonredundant protein (nr) (11), Kyoto Encyclopedia of Genes and Genomes (KEGG) (12), Clusters of Orthologous Groups of proteins (COG) (13), Gene Ontology (GO) (14), and Carbohydrate-Active enZYmes (CAZy) databases (15).

As a result of next-generation sequencing, 20,382,470 clean reads were obtained, with an average length of 148 bp, which were mainly used for correction. PacBio sequencing generated 232,404 sequences with an average length of 3,620 bp and an *N*_50_ value of 4,551 bp. The complete genome was 4,475,991 bp long, with a GC content of 75.35%. Annotation by Prodigal identified 4,074 protein-coding genes and 94 noncoding RNAs in the Y8 genome. A total of 3,872 genes were assigned to the COG functional categories for (i) transport and metabolism of amino acids (261), carbohydrates (424), inorganic ions (186), lipids (82), and coenzymes (105); (ii) transcription (351); (iii) signal transduction (205); (iv) cell wall/membrane biogenesis (164); and (v) general function prediction only (381).

Three copies of the 16S rRNA gene were detected in the Y8 genome. The 16S rRNA gene sequence of Y8 exhibited a high level of similarity to Cellulomonas pakistanensis (99.0%, GenBank accession number NR_125452) and Cellulomonas hominis (98.3%, GenBank accession number NR_029288). We also calculated its average nucleotide identity (ANI) and DNA-DNA hybridization (DDH) values via the ANI calculator (16) (https://www.ezbiocloud.net/tools/ani) and the Genome-to-Genome Distance Calculator (17) (http://ggdc.dsmz.de/ggdc.php) by using the reported draft genome sequence of Cellulomonas pakistanensis (NCBI assembly number ASM131550v1), with default parameter settings, and both of the results obtained (93.03% and 52.5%, respectively) were below the corresponding threshold. This index-based taxonomic assignment of Y8 suggested that it might be a novel species in the genus *Cellulomonas* (18).

### Data availability.

The complete sequence of *Cellulomonas* sp. Y8 has been deposited in GenBank under accession number CP041203 (chromosome), BioProject number PRJNA550281, and SRA accession numbers SRR9639642 (Illumina) and SRR9639643 (PacBio).

## References

[1] McCarthy A 2010 Third generation DNA sequencing: Pacific Biosciences’ single molecule real time technology. Chem Biol 17:675–676. doi:10.1016/j.chembiol.2010.07.004. 20659677

[2] Martin M 2011 Cutadapt removes adapter sequences from high-throughput sequencing reads. EMBnet J 17:3. doi:10.14806/ej.17.1.200.

[3] Eid J, Fehr A, Gray J, Luong K, Lyle J, Otto G, Peluso P, Rank D, Baybayan P, Bettman B, Bibillo A, Bjornson K, Chaudhuri B, Christians F, Cicero R, Clark S, Dalal R, Dewinter A, Dixon J, Foquet M, Gaertner A, Hardenbol P, Heiner C, Hester K, Holden D, Kearns G, Kong X, Kuse R, Lacroix Y, Lin S, Lundquist P, Ma C, Marks P, Maxham M, Murphy D, Park I, Pham T, Phillips M, Roy J, Sebra R, Shen G, Sorenson J, Tomaney A, Travers K, Trulson M, Vieceli J, Wegener J, Wu D, Yang A, Zaccarin D, Zhao P, Zhong F, Korlach J, Turner S 2009 Real-time DNA sequencing from single polymerase molecules. Science 323:133–138. doi:10.1126/science.1162986. 19023044

[4] Goldberg SM, Johnson J, Busam D, Feldblyum T, Ferriera S, Friedman R, Halpern A, Khouri H, Kravitz SA, Lauro FM, Li K, Rogers YH, Strausberg R, Sutton G, Tallon L, Thomas T, Venter E, Frazier M, Venter JC 2006 A Sanger/pyrosequencing hybrid approach for the generation of high-quality draft assemblies of marine microbial genomes. Proc Natl Acad Sci U S A 103:11240–11245. doi:10.1073/pnas.0604351103. 16840556 PMC1544072

[5] Myers EW, Sutton GG, Delcher AL, Dew IM, Fasulo DP, Flanigan MJ, Kravitz SA, Mobarry CM, Reinert KH, Remington KA, Anson EL, Bolanos RA, Chou HH, Jordan CM, Halpern AL, Lonardi S, Beasley EM, Brandon RC, Chen L, Dunn PJ, Lai Z, Liang Y, Nusskern DR, Zhan M, Zhang Q, Zheng X, Rubin GM, Adams MD, Venter JC 2000 A whole-genome assembly of Drosophila. Science 287:2196–2204. doi:10.1126/science.287.5461.2196. 10731133

[6] Berlin K, Koren S, Chin CS, Drake JP, Landolin JM, Phillippy AM 2015 Assembling large genomes with single-molecule sequencing and locality-sensitive hashing. Nat Biotechnol 33:623–630. doi:10.1038/nbt.3238. 26006009

[7] Walker BJ, Abeel T, Shea T, Priest M, Abouelliel A, Sakthikumar S, Cuomo CA, Zeng Q, Wortman J, Young SK, Earl AM 2014 Pilon: an integrated tool for comprehensive microbial variant detection and genome assembly improvement. PLoS One 9:e112963. doi:10.1371/journal.pone.0112963. 25409509 PMC4237348

[8] Delcher AL, Bratke KA, Powers EC, Salzberg SL 2007 Identifying bacterial genes and endosymbiont DNA with Glimmer. Bioinformatics 23:673–679. doi:10.1093/bioinformatics/btm009. 17237039 PMC2387122

[9] Lowe TM, Eddy SR 1997 tRNAscan-SE: a program for improved detection of transfer RNA genes in genomic sequence. Nucleic Acids Res 25:955–964. doi:10.1093/nar/25.5.955. 9023104 PMC146525

[10] Lagesen K, Hallin P, Rodland EA, Staerfeldt HH, Rognes T, Ussery DW 2007 RNAmmer: consistent and rapid annotation of ribosomal RNA genes. Nucleic Acids Res 35:3100–3108. doi:10.1093/nar/gkm160. 17452365 PMC1888812

[11] Pruitt KD, Tatusova T, Maglott DR 2005 NCBI Reference Sequence (RefSeq): a curated non-redundant sequence database of genomes, transcripts and proteins. Nucleic Acids Res 33:D501–D504. doi:10.1093/nar/gki025. 15608248 PMC539979

[12] Kanehisa M, Goto S 2000 KEGG: Kyoto Encyclopedia of Genes and Genomes. Nucleic Acids Res 28:27–30. doi:10.1093/nar/28.1.27. 10592173 PMC102409

[13] Tatusov RL, Koonin EV, Lipman DJ 1997 A genomic perspective on protein families. Science 278:631–637. doi:10.1126/science.278.5338.631. 9381173

[14] Harris MA, Clark J, Ireland A, Lomax J, Ashburner M, Foulger R, Eilbeck K, Lewis S, Marshall B, Mungall C, Richter J, Rubin GM, Blake JA, Bult C, Dolan M, Drabkin H, Eppig JT, Hill DP, Ni L, Ringwald M, Balakrishnan R, Cherry JM, Christie KR, Costanzo MC, Dwight SS, Engel S, Fisk DG, Hirschman JE, Hong EL, Nash RS, Sethuraman A, Theesfeld CL, Botstein D, Dolinski K, Feierbach B, Berardini T, Mundodi S, Rhee SY, Apweiler R, Barrell D, Camon E, Dimmer E, Lee V, Chisholm R, Gaudet P, Kibbe W, Kishore R, Schwarz EM, Sternberg P, Gwinn M, Hannick L, Wortman J, Berriman M, Wood V, de la Cruz N, Tonellato P, Jaiswal P, Seigfried T, White R, Gene Ontology Consortium 2004 The Gene Ontology (GO) database and informatics resource. Nucleic Acids Res 32:D258–D261. doi:10.1093/nar/gkh036. 14681407 PMC308770

[15] Lombard V, Golaconda Ramulu H, Drula E, Coutinho PM, Henrissat B 2014 The carbohydrate-active enzymes database (CAZy) in 2013. Nucleic Acids Res 42:D490–D495. doi:10.1093/nar/gkt1178. 24270786 PMC3965031

[16] Yoon SH, Ha SM, Lim J, Kwon S, Chun J 2017 A large-scale evaluation of algorithms to calculate average nucleotide identity. Antonie Van Leeuwenhoek 110:1281–1286. doi:10.1007/s10482-017-0844-4. 28204908

[17] Meier-Kolthoff JP, Auch AF, Klenk H-P, Göker M 2013 Genome sequence-based species delimitation with confidence intervals and improved distance functions. BMC Bioinformatics 14:60. doi:10.1186/1471-2105-14-60. 23432962 PMC3665452

[18] Kim M, Oh HS, Park SC, Chun J 2014 Towards a taxonomic coherence between average nucleotide identity and 16S rRNA gene sequence similarity for species demarcation of prokaryotes. Int J Syst Evol Microbiol 64:346–351. doi:10.1099/ijs.0.059774-0. 24505072

